# Copper resource trade resilience under the belt and road initiative: Who leads, who lags?

**DOI:** 10.1016/j.isci.2026.116493

**Published:** 2026-06-24

**Authors:** Yibo Wang, Andi Zhang, Jianping Ge

**Affiliations:** 1 School of Economics and Management, China University of Geosciences Beijing, Beijing 10083, China; 2 Institute of Natural Resources Strategic Development, China University of Geosciences Beijing, Beijing 10083, China

**Keywords:** environmental science, natural resources

## Abstract

Amid rising global geopolitical uncertainties and growing resource security risks, establishing a stable, efficient, and sustainable trade system has become a key pillar of the Belt and Road Initiative (BRI). This study develops a multi-dimensional resilience framework and evaluates copper resource trade resilience for 63 Belt and Road countries from 2004 to 2023 using an integrated objective-weighting scheme. The results show a steady overall improvement alongside pronounced and widening disparities between high- and low-resilience countries. Spatially, a “high-resilience core” persists in Central and Eastern Europe, while a “resilience trough” concentrates in West Asia and the Middle East, revealing a durable core-periphery structure. Shapley decomposition indicates that institutional coordination and international engagement account for most of the leader-laggard gap. Exploiting staggered BRI participation, multi-period DID evidence suggests that the BRI causally strengthens trade resilience, with asymmetric and timing-dependent gains. These results inform more targeted, equity-oriented BRI policy design.

## Highlights

• Developed a three-capability framework for copper resource trade resilience
• Trade Resilience improved overall, but widened into a core-periphery structure
• Adjustment and recovery capacity drives most leader-laggard resilience gaps
• The impact of BRI on trade resilience and regional synergy is unbalanced

## Graphical abstract

**Figure:**
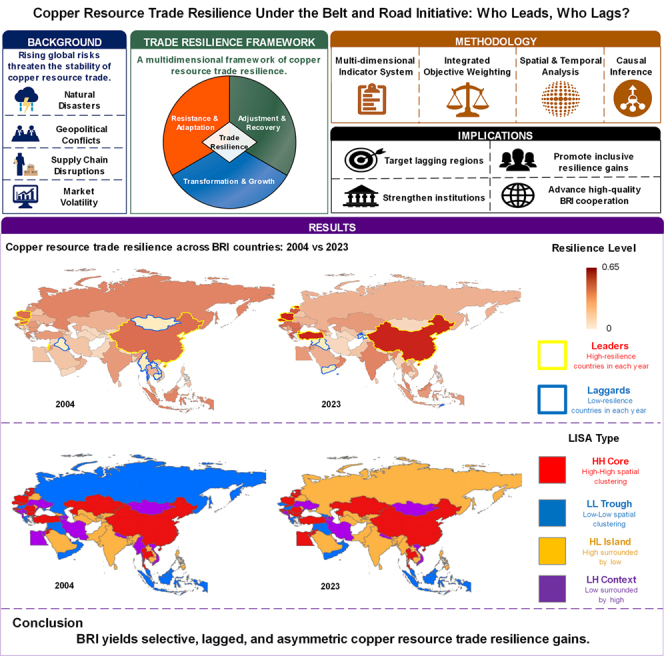

## Introduction

Copper resources constitute a critical material foundation for the global industrial economy and the transition toward green and low-carbon development, playing a pivotal role in emerging sectors such as new energy vehicles, aerospace, and computing-communication industries.^1^^,^^2^^,^^3^ They have thus become a strategic mineral resource contested by nations worldwide.^4^ However, the global distribution of copper resources is highly uneven; copper ore reserves are predominantly concentrated in South America and Africa (nearly 50%), whereas refining capacity and market demand are largely centered in Asia and Europe (over 65%).^3^^,^^5^^,^^6^ This supply-demand mismatch has driven the expansion of international copper resource trade.^7^ In recent years, intensifying global economic volatility, frequent geopolitical conflicts, and repeated disruptions to international supply chains have increasingly highlighted the issues of stability and security within resource product trade systems. Consequently, trade “resilience” has attracted widespread attention from both policymakers and the academic community.^8^^,^^9^

The concept of “resilience,” originating in ecology and engineering, refers to a system’s ability to resist, recover, and adapt to external shocks.^10^ In recent years, this concept has been applied in economics to analyze the stability and recovery of economic systems under external risks, leading to the development of economic resilience, supply chain resilience, and trade resilience.^11^^,^^12^^,^^13^^,^^14^ Trade resilience refers to the ability of a country or region to maintain, restore, and rebuild stable foreign trade in the face of external shocks, particularly for strategically important critical minerals such as copper.^13^ While scholars have developed indicator systems for measuring trade resilience, most existing research relies on regional or economic resilience frameworks and simplifies the complexity of trade systems by using single indicators such as export complexity or trade dependence.^13^^,^^14^^,^^15^^,^^16^^,^^17^^,^^18^^,^^19^ Although this method is useful, it has not fully captured the diversified adjustment of trade resilience and the path of capacity enhancement, thus limiting the scope and explanatory power of existing research. This study, therefore, adopts a more comprehensive, multidimensional approach to evaluate trade resilience. Specifically, trade resilience is conceptualized as a composite capability encompassing resistance and adaptation, adjustment and recovery, and transformation and growth, and is operationalized through a three-dimensional indicator system that structurally reflects these mechanisms.

Since its launch in 2013, the Belt and Road Initiative (BRI) has facilitated resource complementarity and trade cooperation, particularly in copper trade.^20^ China, the world’s largest copper importer, depends on external sources for over 70% of its copper, while countries such as Kazakhstan and Indonesia hold significant potential as copper exporters.^21^ While existing studies have examined the BRI’s effects on trade volumes, trade cost reductions, and trade liberalization,^22^^,^^23^^,^^24^^,^^25^ research on how the BRI affects trade resilience, particularly in critical minerals such as copper, remains underexplored. Furthermore, while the BRI has led to significant changes in the scale and structure of copper trade, its impact on trade resilience, particularly in enhancing spatial optimization and regional synergy, has not been systematically studied. In this study, regional synergy refers to the coordinated enhancement of trade resilience across countries through policy alignment, institutional compatibility, and spatial spillover effects, rather than isolated improvements at the national level. This research fills this gap by developing a multidimensional resilience framework to evaluate the copper resource trade resilience of BRI countries and exploring how the BRI enhances regional synergy.

The improvement in trade resilience is reflected not only in the enhancement of specific national indicators but, more importantly, in the emergence of regional spatial coordination under policy-driven integration. This results in the evolution of spatial agglomeration patterns from “dispersed-isolated” to “clustered-synergistic.”^26^^,^^27^^,^^28^ Although national resilience exhibits pronounced spatial disparities among the Belt and Road countries,^17^^,^^18^ the mechanisms underlying these differences, particularly in terms of the BRI’s influence on enhancing regional synergy in trade resilience, remain underexplored. This study addresses this gap by systematically investigating the spatial coordination effects of BRI policies and the role of policy coordination in improving trade resilience among BRI countries.

Compared with existing literature, this study advances research on critical mineral trade resilience and the BRI in three respects. First, it develops a mechanism-based conceptual framework for copper resource trade resilience and operationalizes it through a three-dimensional indicator system, enabling a structured decomposition of resilience into resistance and adaptation, adjustment and recovery, and transformation and growth capacities. This approach moves beyond single-indicator or purely outcome-based measurements and captures the internal structure of trade resilience. Second, rather than treating spatial analysis as an end, this study employs spatial econometric tools to reveal how structural differences in trade resilience are externalized in spatial patterns, thereby identifying the formation and evolution of core-periphery structures among Belt and Road countries. Third, by combining spatial analysis with a difference-in-differences framework, this study provides causal evidence on how the BRI reshapes regional synergy in copper resource trade resilience, clarifying the policy mechanisms by which coordination, institutional alignment, and spatial interaction jointly influence resilience outcomes.

### Analytical framework for copper resource trade resilience

#### Definition, connotation, and specificity of copper resource trade resilience

Copper resource trade resilience refers to the capacity of a country or region to maintain the stability, continuity, and developmental trajectory of its international trade in mineral resources when confronted with external shocks (e.g., price fluctuations, supply chain disruptions, policy changes).^29^ Extending from the general concept of trade resilience, copper resource trade resilience encompasses three dimensions: resistance and adaptation capacity, adjustment and recovery capacity, and transformation and growth capacity.^30^^,^^31^ Resistance and adaptation capacity refers to the ability to sustain mineral resource trade activities under external shocks; Adjustment and recovery capacity denotes the ability to swiftly restore and realign trade activities to their pre-shock levels after a disruption; Transformation and growth capacity signifies the ability to achieve sustained growth and optimization of mineral resource trade activities over the long term through technological innovation, market expansion, and related measures. Given copper’s non-renewable nature and uneven geographical distribution, its trade activities are subject to greater uncertainty. Compared with general commodities, copper resource trade resilience requires greater emphasis on supply chain stability, market demand volatility, and changes in the policy environment.^21^

#### Generative logic of copper resource trade resilience

**Figure 1:**
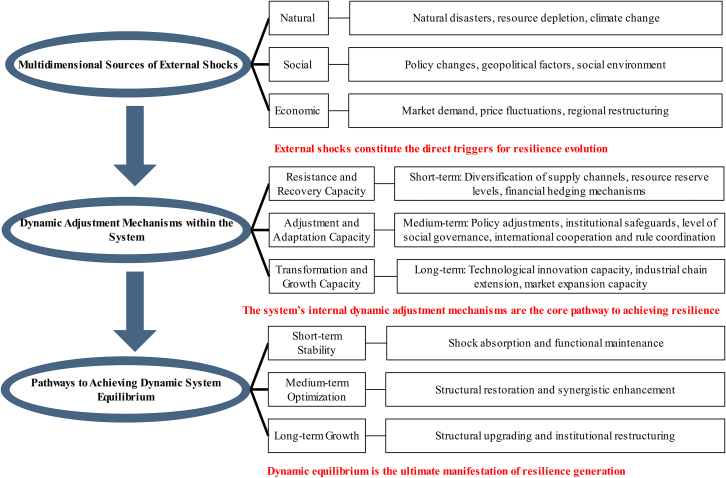
Generative logic of copper resource trade resilience: short-term, medium-term, and long-term stages of recovery, adjustment, and transformation

The generative logic of copper resource trade resilience examines the intrinsic mechanisms by which a trade system maintains stability, continuity, and developmental capacity under external shocks. In essence, it reflects the system’s ability to achieve equilibrium and adaptation through dynamic adjustment in response to external disturbances.^32^^,^^33^ As shown in Figure 1, this process can be summarized as a three-stage logic of “external shocks-system adjustment-dynamic equilibrium,” emphasizing the system’s capacity to recover from disturbances and achieve self-evolution. First, external shocks constitute the direct triggers for resilience evolution. Copper resource trade relies on global cross-border transportation and a highly internationalized institutional environment, making it vulnerable to a wide array of factors, including natural disasters (e.g., extreme weather events and geological hazards), socio-political incidents (e.g., sanctions, conflicts), and economic fluctuations (e.g., sharp price volatility and regional market restructuring). These shocks affect the system via mechanisms such as supply interruptions, demand disruptions, and the suspension of trade routes, leading to deviations from normal operational states.^34^^,^^35^ Second, the system’s internal dynamic adjustment mechanisms represent the core pathway for resilience realization. Following a shock, the system leverages its inherent capacities to implement multi-level responses: In the short term, mitigating impacts through trade partner diversification, strategic reserves, and financial hedging mechanisms; in the medium term, restoring and optimizing structural configurations via policy adjustments, institutional safeguards, and international cooperation; and in the long term, achieving structural upgrades through technological innovation, industrial chain extension, and market expansion. Capacities across these dimensions are temporally staged in a gradient sequence, collectively shaping the system’s adaptability and plasticity.^13^^,^^14^^,^^33^ Third, dynamic equilibrium represents the ultimate manifestation of resilience generation. Through hierarchical adjustments in internal mechanisms, the system can achieve stable operation under new environmental conditions, progressing from short-term shock absorption to medium-term structural optimization, and ultimately to long-term growth.^32^ More importantly, this process is not a one-off repair; rather, it operates through positive feedback loops that continuously enhance the system’s coping capacity, forming a cyclical process of “shock-adjustment-upgrading.^36^ Therefore, the generation of copper resource trade resilience is not merely a passive absorption of external shocks, but rather an active adaptation constructed through the synergistic integration of multidimensional capacities in a complex environment.

#### Theoretical framework of copper resource trade resilience

**Figure 2:**
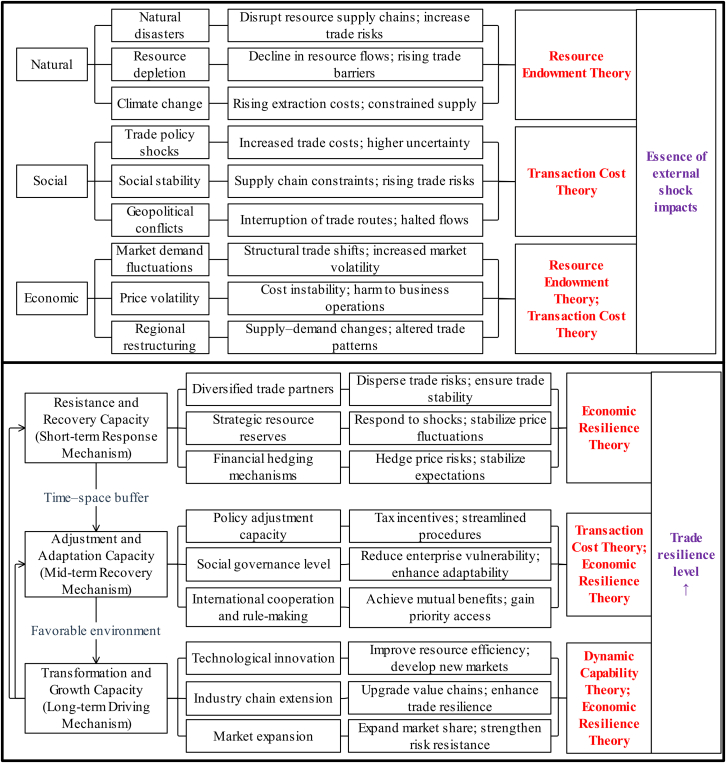
Theoretical framework of copper resource trade resilience: interactions between external shocks and internal system responses

To systematically elucidate the formation mechanism of copper resource trade resilience, this study integrates multiple theoretical perspectives, including resource endowment theory, transaction cost theory, dynamic capability theory, and economic resilience theory, to construct a theoretical framework for copper resource trade resilience. As shown in Figure 2, the framework conceptualizes the copper resource trade system as an open and complex system, wherein the generation of resilience depends on the interaction between the risk pressure of external shocks and the dynamic responses of internal system capabilities.

External shocks serve as the backdrop for trade risk formation. Due to its heavy reliance on natural resources, institutional frameworks, and global markets, the copper resource trade system is highly susceptible to various external shocks. These include natural disruptions, logistical barriers, and climate change (natural shocks); geopolitical instability and trade restrictions (social shocks); and price volatility and market restructuring (economic shocks). These shocks challenge the stability of the trade system and require resilience to emerge. Resource endowment theory highlights how uneven resource distribution amplifies shock uncertainty, while transaction cost theory suggests that such shocks increase transaction uncertainty and enforcement costs, forcing the system to find a new equilibrium.^37^^,^^38^

The internal response mechanisms of the system represent its resilience capabilities in the face of external shocks. These are categorized into three dimensions: resistance and adaptation capacity, adjustment and recovery capacity, and transformation and growth capacity.^30^ Resistance and adaptation capacity involves short-term responses such as diversifying trade partners and implementing risk-sharing mechanisms. Adjustment and recovery capacity pertain to medium-term responses, including institutional adaptation and policy stability. Finally, transformation and growth capacity reflect long-term strategies, such as technological innovation and market expansion. These responses are explained by transaction cost theory (institutional efficiency), economic resilience theory (shock resistance and recovery), and dynamic capability theory (long-term structural evolution), together forming the foundation for copper resource trade resilience.^38^^,^^39^^,^^40^

### Indicator system for copper resource trade resilience

**Figure 3:**
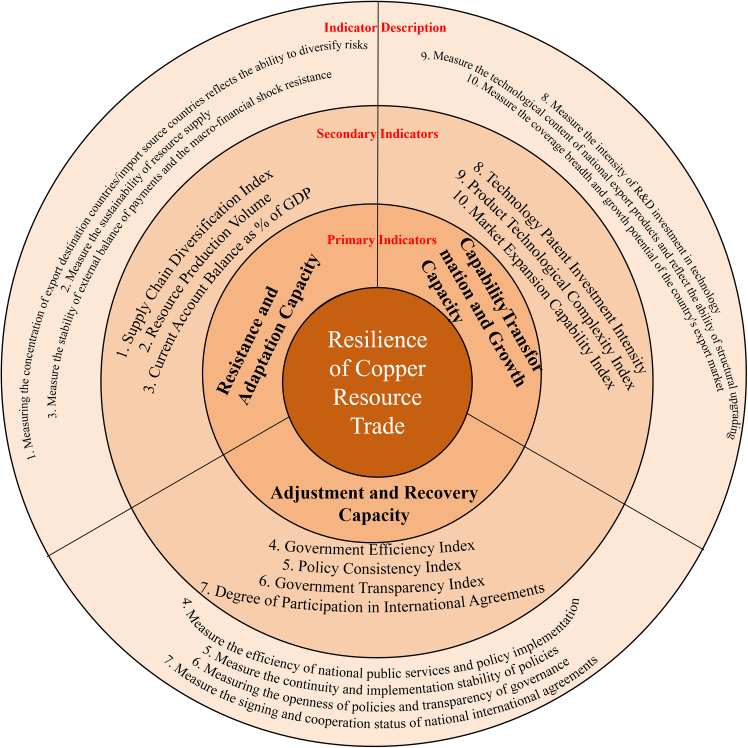
Indicator system for copper resource trade resilience

To comprehensively capture the resilience performance of Belt and Road countries in copper resource trade, this study, based on the aforementioned theoretical framework, develops a composite evaluation index system comprising 10 secondary indicators under three capability dimensions. The indicators are derived from dimensions such as supply chain diversification, resource security capacity, external financial stability, institutional and policy environment, technological innovation capacity, and market expansion potential. This system reflects both macro-structural factors and micro-level soft elements such as institutional and technological capabilities. The specific indicators are shown in Figure 3.

## Results

This section presents a comprehensive set of empirical results on the evolution, differentiation, spatial organization, and policy effects of copper resource trade resilience among Belt and Road countries. The results are structured to progressively address four interrelated questions. First, the temporal evolution analysis reveals a long-term upward trend in trade resilience accompanied by persistent and widening cross-country differentiation, with distinct phases of adjustment, acceleration, and high-level consolidation. Second, the ranking and structural decomposition analyses identify a stable leader-laggard configuration, showing that resilience differences are primarily driven by institutional adjustment and recovery capacity rather than resource endowment alone. Third, the spatial analysis demonstrates that these structural differences are increasingly externalized into a core-periphery pattern, characterized by persistent high-resilience cores and low-resilience troughs, alongside fragmented and limited diffusion effects. Finally, the causal analysis based on a difference-in-differences framework provides robust evidence that the BRI has significantly enhanced copper resource trade resilience, particularly for early and structurally capable participants, and has reinforced selective structural coordination and spatial synergy rather than uniform convergence.

### Temporal evolution of copper resource trade resilience

#### Overall evolution trend of copper resource trade resilience

To reveal the overall development trend and structural distribution characteristics of copper resource trade resilience among the Belt and Road countries, this study analyzes the measured results of copper resource trade resilience from 2004 to 2023 based on annual statistical descriptions.

**Figure 4:**
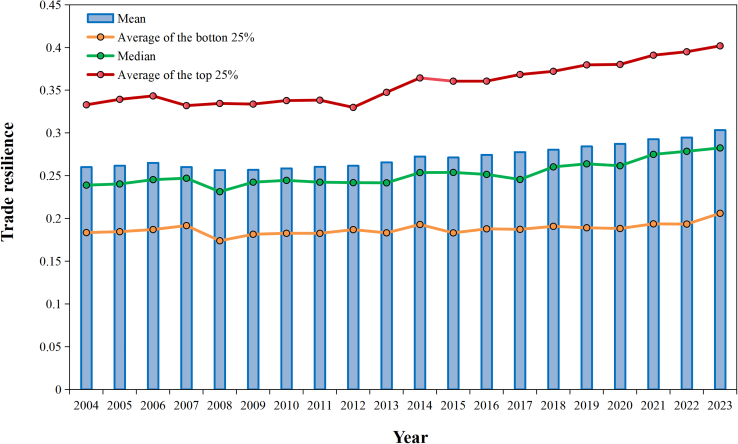
Trend of trade resilience among the Belt and Road countries from 2004 to 2023

As shown in Figure 4, the overall evolution of copper resource trade resilience across Belt and Road countries exhibits a pattern of “steady improvement accompanied by persistent structural differentiation.”

First, the overall level of trade resilience shows a gradual upward trend. From 2004 to 2023, the mean value of copper resource trade resilience increases steadily, indicating a continuous enhancement in the stability and adaptive capacity of the copper resource trade system among Belt and Road countries in response to external shocks and structural changes. This upward trajectory becomes more pronounced after the mid-2010s.

Second, improvements in trade resilience are unevenly distributed across countries. Although the mean level rises steadily over time, the median remains consistently below the mean throughout the study period, while the average resilience level of the bottom 25% of countries exhibits only limited growth. This divergence suggests a right-skewed distribution of trade resilience, whereby a subset of countries with relatively high resilience levels exerts a strong upward influence on the overall mean. In contrast, the majority of countries experience more modest improvements, indicating that broad-based convergence in trade resilience has not yet been achieved.

Third, inter-country differentiation in trade resilience has gradually intensified. As shown by the widening gap between the top 25% and bottom 25% of countries, disparities in trade resilience begin to expand noticeably after 2008 and persist throughout the subsequent period. While high-resilience countries continue to improve steadily, the resilience of low-resilience countries remains comparatively stagnant, reinforcing a structural divide between leading and lagging economies in terms of copper resource trade resilience.

Notably, after 2013, the mean trade resilience showed a noticeable upward trend, suggesting that the BRI policy may have had a positive impact on enhancing the trade adaptation capacity of marginal countries and narrowing the resilience gap, exerting significant supporting effects in some developing economies. However, breakthroughs in trade resilience among low-ranking countries have not yet occurred, reflecting the imbalance in development trends among countries.

#### Phased evolution characteristics of copper resource trade resilience

Building on the analysis of the overall evolution trend, this study further examines the phased evolution characteristics of copper resource trade resilience by employing the three-year moving average growth rate of the resilience index. The moving average method smooths short-term fluctuations and highlights sustained trends by reducing the influence of temporary shocks, thereby allowing a clearer identification of medium to long-term changes in trade resilience dynamics. A three-year window is adopted to balance sensitivity to recent changes and robustness against short-term volatility, a commonly used approach in time-series analysis of economic resilience and regional development^.^^41^^,^^42^

To identify different growth stages, the interquartile range (IQR) approach is applied to the three-year moving average growth rate series.^43^ Based on the empirical distribution of the growth rates from 2007 to 2023, the first quartile (Q1), median (Q2), and third quartile (Q3) are estimated at 0.51%, 1.07%, and 1.21%, respectively. These thresholds indicate that the growth rate distribution is asymmetric and moderately right-skewed, with most observations concentrated in the medium-growth range and relatively fewer years exhibiting exceptionally high or negative growth. Rather than mechanically assigning each year to a quartile group, these quantile thresholds are used as reference benchmarks to support the identification of broader evolutionary phases in combination with the temporal trajectory of the growth rate.

**Figure 5:**
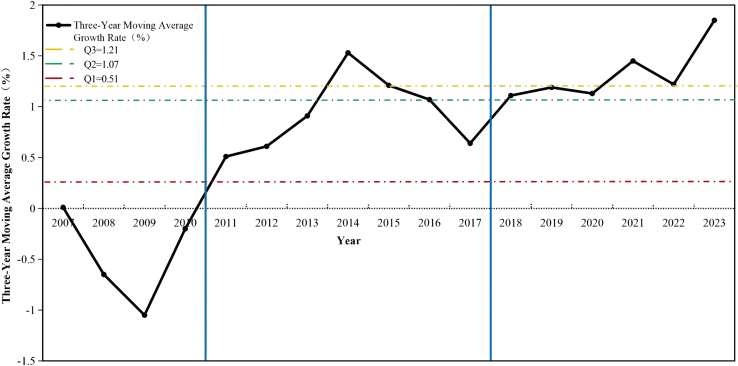
Phase diagram of copper resource trade resilience, three-year moving average growth rate based on the IQR method

As shown in Figure 5, the three-year moving average growth rate of copper resource trade resilience exhibits a clear pattern of phased evolution, which can be summarized into three major stages.

First, the period from 2007 to 2010 represents an initial adjustment phase of trade resilience. During this stage, copper resource trade resilience remained at a relatively low level, characterized by slow improvement, pronounced volatility, and limited differentiation among countries. The three-year moving average growth rate frequently stayed below the Q1 threshold and even turned negative in some years, reflecting the fragility of the trade system at an early stage of development. This pattern can be attributed to the strong dependence of many Belt and Road countries on external markets, coupled with weak domestic resource development capacity and insufficient market expansion capabilities.^44^ In addition, policy coordination and governance frameworks across countries were still unstable, and regional cooperation mechanisms had not yet formed. Although the global financial crisis in 2008 exerted a strong shock on international commodity markets and supply chains, its impact on copper resource trade resilience during this phase was largely short-term and cyclical, resulting mainly in heightened fluctuations rather than a structural break.

Second, the period from 2011 to 2017 can be identified as a phase of accelerated improvement with pronounced heterogeneity. In this stage, copper resource trade resilience exhibited a clear upward trajectory, with the growth rate gradually rising from the lower-quartile range to reach a temporary peak in the mid-2010s. Notably, the three-year moving average growth rate exceeded the Q3 benchmark in 2014 and remained at relatively high levels in the subsequent years before retreating toward the median range by 2017. This pattern indicates that the resilience system experienced a phase of rapid strengthening, followed by partial consolidation. The acceleration of trade resilience during this period was accompanied by increasing differentiation among countries, as leading economies advanced more rapidly while less competitive countries improved at a slower pace. The emergence of large-scale regional cooperation initiatives after 2013, together with improvements in cross-border connectivity and institutional coordination, coincided with this acceleration phase and provided a supportive external environment for enhancing copper resource trade resilience.^44^ However, the decline in growth momentum toward the end of this stage suggests that such improvements were not uniformly sustained across countries.

Third, the period from 2018 to 2023 represents a high-level enhancement phase under intensified external uncertainty. During this phase, copper resource trade resilience was maintained at a relatively high level overall, with the three-year moving average growth rate consistently remaining above the Q2 threshold. The system displayed stronger internal stability compared with earlier periods, indicating that the foundational capacity of copper resource trade resilience had been substantially reinforced. At the same time, the growth pattern in this stage was not linear. While the resilience system continued to strengthen steadily in the early years of the phase, renewed acceleration became evident after 2020, with multiple years exceeding the Q3 benchmark and reaching a new high by 2023. This evolution occurred against the backdrop of heightened external shocks, including global supply chain disruptions, geopolitical tensions, and market volatility. High-resilience countries were generally able to absorb these shocks and maintain upward momentum through institutional flexibility and diversified trade structures, whereas low-resilience countries faced increasing constraints, leading to further structural differentiation within the Belt and Road region.

#### Who leads, who lags

**Figure 6:**
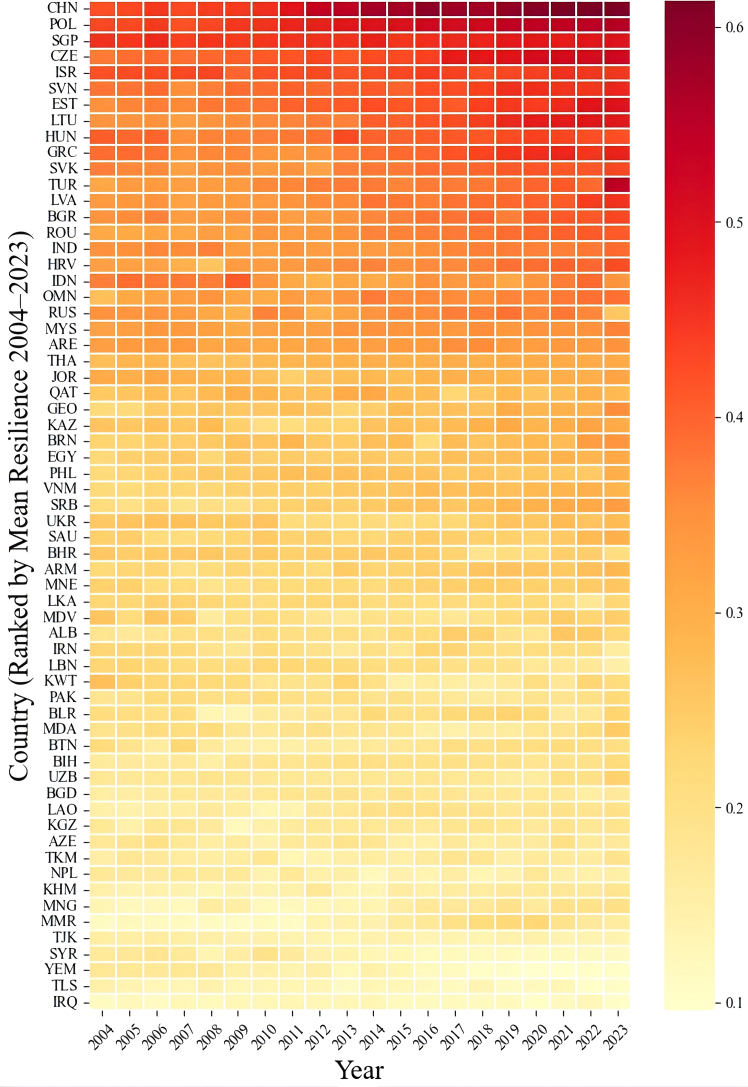
Temporal distribution of copper resource trade resilience among the Belt and Road countries Notes: The abbreviated names of countries are listed in Table S1.

To further identify the long-term differentiated pattern of copper resource trade resilience among Belt and Road countries, this study examines cross-country ranking dynamics over 2004-2023, as shown in Figure 6. Overall, countries can be broadly characterized by three persistence profiles: “high position lock-in,” “mid-position oscillation,” and “low-position lagging,” highlighting a stable yet stratified resilience structure. Notably, this stratification is not merely a reflection of resource endowment; it is also consistent with differences in institutional enforcement, market responsiveness, and countries’ capacity to participate in and adapt to evolving global governance arrangements. The scores of copper resource trade resilience indicators for all countries from 2004 to 2023 are shown in Table S2.

Locked at the Top, Stuck at the Bottom: Two-Decade Ranking Dynamics.

First, leading countries exhibit strong persistence, forming a clear “high position lock-in” pattern. Based on the 2004-2023 country mean of copper resource trade resilience, the top group comprises China (0.527), Poland (0.486), Singapore (0.462), Czechia (0.441), and so forth. These countries jointly constitute a relatively stable upper tier: Their positions are consistently concentrated in the top quartile over the entire period, with only limited short-term reordering within the group. For example, China’s resilience level rises from 0.423 (2004) to 0.613 (2023), and its ranking remains in the very top throughout the period (top-three range), reflecting a persistent advantage in sustaining and upgrading copper resource trade resilience.

Second, mid-ranking countries display noticeably higher mobility and internal differentiation. Figure 6 suggests that a substantial share of countries remain within an intermediate band but experience recurrent rank reshuffling, indicating that resilience improvements are neither uniformly stable nor structurally guaranteed. Importantly, the mid-tier contains both “upward movers” and “downward drifters.” As a typical upward mover, Turkiye shows a marked improvement from rank 20 (2004) to rank 3 (2023), with its resilience level increasing from 0.311 (2004) to 0.542 (2023), demonstrating a clear late-period strengthening pattern. Similarly, Georgia improves from rank 40 (2004) to rank 20 (2023) (0.222 to 0.358), and Serbia improves from rank 42 (2004) to rank 24 (2023) (0.219 to 0.333), reflecting gradual catching-up dynamics. In contrast, some countries exhibit downward movement over time; for instance, the Russian Federation declines from rank 11 (2004) to rank 36 (2023), with resilience decreasing from 0.352 (2004) to 0.259 (2023), indicating that early advantages were not maintained in the later period.

Third, the lagging group remains persistently constrained, forming a “low-position lagging” pattern with limited upward mobility. The bottom of the distribution, based on the 2004-2023 mean resilience, includes Syria (0.144), Yemen (0.139), Timor-Leste (0.130), and Iraq (0.123), and so forth. The structural persistence of the bottom tier is notable: Several countries remain in the bottom quartile for nearly the entire period, and some exhibit further deterioration toward the end of the sample. For example, Iraq stays at the bottom consistently (mean 0.123), and Yemen drops from rank 49 (2004) to rank 63 (2023), with resilience decreasing from 0.178 (2004) to 0.107 (2023). These patterns collectively indicate that downward rigidity and weak mobility are salient features of the low-resilience tier.

Overall, the ranking distribution demonstrates pronounced stratification. The average resilience of the top 10 countries is 0.438, compared with 0.148 for the bottom 10, yielding a gap of 0.290 (approximately a 3-fold difference). In 2023, the gap further widens: The top 10 average reaches 0.497, whereas the bottom 10 average remains at 0.149, reinforcing the persistence of cross-country divergence.

#### Why leaders lead, why laggards lag: A shapley-based structural explanation

Building on the ranking dynamics in Figure 6, this section explains cross-country divergence through a two-layer structural decomposition. Specifically, the copper resource trade resilience gap between the top 10 and bottom 10 countries is decomposed into three capability sub-indices and ten indicator-level components. The result of Shapley’s two-layer structural decomposition are shown in Table S3.

**Figure 7:**
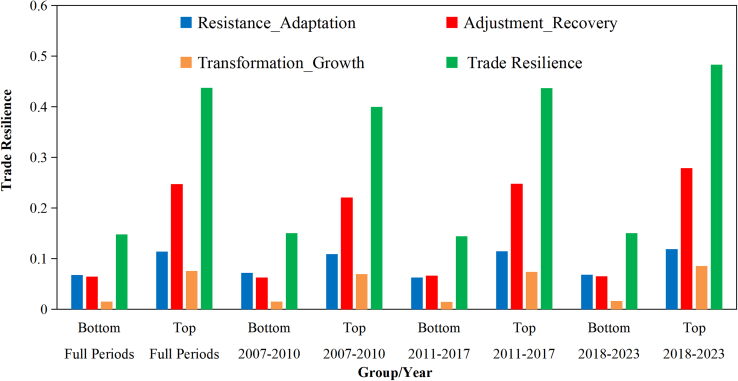
Three capability decomposition of differences in copper resource trade resilience between the top 10 and the bottom 10 countries at different stages

Capability-layer decomposition. The copper resource trade resilience gap is primarily an “Adjustment and Recovery Capacity” gap. Across the full period, the mean resilience of the top 10 countries is 0.4377, compared with 0.1482 for the bottom 10 countries, yielding a total gap of 0.2895. This gap is strongly dominated by the Adjustment and Recovery capability. As shown in Figure 7, the adjustment and recovery capability gap is 0.1828, accounting for 63.14% of the total resilience gap; the resistance and adaptation capability gap is 0.0462, accounting for 15.96%; the transformation and growth capability gap is 0.0605, accounting for 20.90%. This pattern is stable across stages. In 2007-2010, 2011-2017, and 2018-2023, Adjustment and Recovery capability consistently explains approximately 62-64% of the top-bottom gap (63.20%, 62.12%, and 64.13%, respectively), while Transformation and Growth capability contributes around 20-22% and Resistance and Adaptation capability around 15-18%. Moreover, the overall top-bottom gap expands over time, from 0.2495 (2007-2010) to 0.2924 (2011-2017) and further to 0.3329 (2018-2023), indicating a persistent and widening structural divergence rather than a transient fluctuation. The above evidence implies that long-term leadership in copper resource trade resilience is more about systematic advantages in institutional adjustment, policy coordination, and recovery capacity, which cumulatively shape the ability to stabilize trade systems, reconfigure linkages, and sustain functioning under shocks.

**Figure 8:**
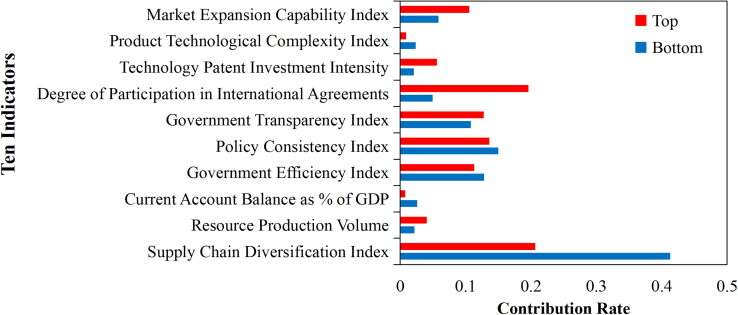
Decomposition of 10 indicators contributing to the differences in copper resource trade resilience between the top 10 and bottom 10 countries

Indicator-layer decomposition, institutional participation, and governance quality are the principal separators. At the indicator level, the top-bottom trade resilience gap (0.2895) can be further attributed to specific components. The largest contributors are concentrated in institutional participation and governance-related indicators, as well as market expansion capability, as shown in Figure 8. Degree of Participation in International Agreements gap 0.0775, contributing 26.78% of the total gap; Government Transparency gap 0.0391, contributing 13.52%; Market Expansion Capability gap 0.0389, contributing 13.42%; Policy Consistency gap 0.0362, contributing 12.52%; Government Efficiency gap 0.0299, contributing 10.33%. Together, these five factors explain approximately 76.6% of the top-bottom trade resilience gap, underscoring that the leader-laggard separation is primarily institutional and capability driven. In contrast, resource-side factors contribute more moderately. Notably, Current Account Balance as% of GDP has a near-zero contribution (gap 0.0003), suggesting that it does not systematically differentiate leaders from laggards in this sample. Similarly, Product Technological Complexity contributes only marginally (a gap of 0.0007), indicating that the observed cross-country divergence is not primarily driven by this component at the aggregate level.

Overall, the leader-laggard divide in copper resource trade resilience is best characterized as a persistent and widening structural divergence, mainly driven by Adjustment and Recovery capacity, and, at the indicator level, by international institutional participation, governance quality, policy consistency, and market expansion capability, rather than by resource endowment alone. This evidence provides a direct structural explanation for the “high position lock-in” and “low-position lagging” patterns observed in Figure 6.

### Who aggregates as core, and Who Falls to the periphery

#### Identification of spatial clustering of copper resource trade resilience

To reveal the spatial structural evolution of copper resource trade resilience among the Belt and Road countries, this chapter combines global and local spatial autocorrelation methods to conduct time-series analyses using Moran’s I and LISA. The dual perspective is used to identify spatial clustering, polarization trends, and core-periphery patterns of trade resilience. The results of Moran’s I and LISA are shown in Tables S4 and S5, respectively.

**Figure 9:**
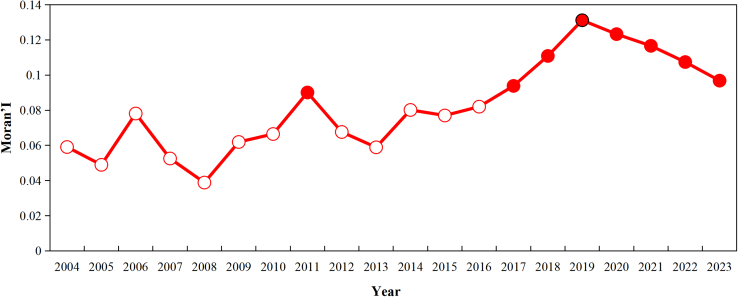
Trend of the spatial autocorrelation index of copper resource trade resilience from 2004 to 2023 Notes: Hollow circles represent non-significant, solid circles without outline represent ∗*p* < 0.10, and solid circles with black outline represent ∗∗*p* < 0.05.

Moran’s I is used to reflect the overall spatial autocorrelation of copper resource trade resilience. Higher values indicate stronger spatial clustering among high-resilience or low-resilience countries, while greater statistical significance indicates a more stable spatial structure.27 As shown in Figure 9, the Moran’s I values remain positive throughout 2004-2023 (0.039-0.131), indicating that copper trade resilience exhibits a weak-to-moderate positive spatial autocorrelation rather than a purely random spatial pattern. Importantly, the strength and significance of spatial dependence exhibit a clear three-stage evolution.

First (2004-2010), spatial dependence is weak and statistically non-robust. Moran’s I fluctuates at relatively low levels (around 0.039-0.078), and most years are statistically non-significant (*p* > 0.10). Although 2006 approaches marginal significance (*p* = 0.109), the overall evidence suggests that spatial clustering had not yet become a stable structural feature during this phase. In other words, spatial responses were present but fragmented, and resilience differences were not yet spatially organized into a persistent regional pattern. Second (2011-2017), spatial dependence gradually strengthens and begins to show marginal significance. Moran’s I rise to a higher band (about 0.059-0.094), and borderline significance emerges in key years (e.g., 2011 *p* = 0.091; 2017 *p* = 0.089). Meanwhile, 2014-2016 display elevated Moran’s I levels with *p* values close to, though still above 0.10 (0.113-0.104), suggesting an incipient clustering tendency that is not yet fully robust. Taken together, this stage indicates that spatial association in copper trade resilience was forming and consolidating, consistent with the intensifying cross-country differentiation identified in the temporal and ranking analyses. Third (2018-2023), spatial clustering becomes more pronounced, peaks, and then shows mild attenuation. Moran’s I increased substantially in 2018-2019 (0.111-0.131), with 2019 achieving conventional significance (*p* = 0.039). In 2020-2021, Moran’s I remains at a relatively high level (0.123 and 0.117), while *p* values (0.057 and 0.055) are close to the 0.05 threshold, indicating borderline statistical significance and suggesting that clustering effects remained strong but were sensitive to uncertainty. After 2021, Moran’s I declines gradually, yet it stays positive and is still marginally significant at the 10% level in 2022-2023. This pattern implies that spatial dependence did not disappear; rather, the spatial structure entered a phase of high-level adjustment under intensified external shocks and volatility.

Overall, the evolution of Moran’s I suggests that the spatial pattern of copper resource trade resilience became increasingly organized over time, with the most pronounced clustering emerging in the late period. This global evidence is consistent with the “high-position lock-in” and “low-position lagging” structure identified in the ranking analysis, indicating that structural differentiation tends to be spatially externalized.

#### Identification of local spatial heterogeneity in copper resource trade resilience

**Figure 10:**
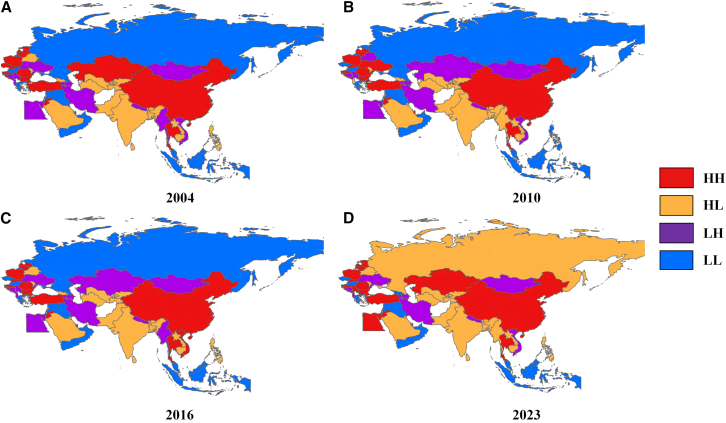
Distribution of four LISA cluster types of countries along the Belt and Road in 2004, 2010, 2016, and 2023

Compared to the overall spatial autocorrelation trend presented by Moran’s I, LISA analysis further reveals the specific role differentiation of the Belt and Road countries in the spatial structure of copper resource trade resilience.^26^ LISA classifies the spatial clustering of individual countries into four types. Figure 10 presents the LISA cluster maps for 2004, 2010, 2016, and 2023, illustrating the evolution of these local spatial configurations over time.

Overall, the LISA results indicate that the spatial structure of copper resource trade resilience is characterized by persistent core-periphery polarization combined with limited and uneven diffusion effects. Two dominant spatial patterns can be identified.

The first is a stable polarized structure, reflected in the long-term coexistence of HH and LL clusters. HH-type countries form a relatively stable “high-resilience core,” mainly concentrated in East Asia and Central-Eastern Europe. Typical HH countries include China, Poland, Czechia, and Slovakia, all of which consistently occupy the HH quadrant in multiple periods, particularly after 2010. These countries not only exhibit high levels of trade resilience individually, but also tend to be spatially proximate to other high-resilience countries, forming contiguous high-value clusters by 2016 and 2023. This pattern suggests that strong institutional capacity, diversified trade structures, and effective adjustment and recovery mechanisms are mutually reinforcing at the regional level. In contrast, LL type countries constitute a persistent “low-resilience trough,” primarily located in West Asia, the Middle East, and parts of South Asia. Countries such as Yemen, Iraq, and Syria remain in the LL quadrant across most observed years, indicating long-term spatial lock-in at low-resilience levels. The spatial continuity of LL clusters implies that low-resilience conditions are not isolated but tend to be regionally reinforced, reflecting compounded constraints related to institutional fragility, limited market integration, and weak adaptive capacity.

The second spatial pattern is a fragmented and unstable interaction structure, reflected in the alternation between HL and LH types. HL-type countries, characterized by high resilience surrounded by low-resilience neighbors, appear repeatedly in the spatial distribution. A representative example is India, which exhibits relatively high trade resilience but is often surrounded by lower-resilience neighboring countries, resulting in a persistent HL configuration. This indicates that high internal resilience does not automatically translate into positive spillovers for neighboring countries. Similarly, some resource-rich economies in Central Asia display HL characteristics in certain years, suggesting that their resilience advantages are largely internally driven rather than regionally diffused. Conversely, LH-type countries occasionally benefit from proximity to high-resilience neighbors but rarely achieve sustained upgrading. Mongolia, for instance, appears as an LH-type country in some periods due to its geographic proximity to high-resilience economies such as China and Russia. However, this spatial advantage has not translated into a stable transition toward HH status, indicating that external proximity alone is insufficient to generate durable improvements in trade resilience without corresponding enhancements in institutional and adjustment capacities.

Taken together, the LISA results demonstrate that the spatial evolution of copper resource trade resilience is dominated by polarization with selective and incomplete diffusion. High-resilience cores and low-resilience peripheries have become increasingly entrenched, while spatial spillover effects remain limited and contingent. This spatial pattern is consistent with the structural decomposition results in Section who leads, who lags, which highlight the central role of institutional adjustment and recovery capacity in shaping resilience differences. Consequently, local spatial clustering reflects not only geographic proximity, but also deeper structural asymmetries in governance capacity and market integration among Belt and Road countries.

### Has the BRI enhanced synergy

#### Causal effects of the BRI on copper resource trade resilience

To further assess whether the BRI has exerted a causal impact on copper resource trade resilience, this study applies a DID framework to the panel data of Belt and Road countries. The estimation results, summarized in Table S6, include baseline fixed-effects DID regressions, dynamic event study specifications, heterogeneity analyses based on the timing of BRI participation, and a series of robustness checks.

The baseline DID estimates indicate a positive and statistically significant average treatment effect of the BRI on copper resource trade resilience. The coefficient on the BRI exposure variable is estimated at 0.0063 and is statistically significant at the 10% level, suggesting that, on average, participation in the BRI is associated with an increase of approximately 0.6 percentage points in the standardized trade resilience index. Although the contemporaneous effect is modest, it provides initial evidence that the BRI contributes positively to resilience enhancement.

When lagged policy effects are incorporated, the estimated impacts become stronger and more precisely identified. The one-period lag of BRI exposure yields a coefficient in the range of 0.012-0.014 (significant at the 1% level), while the two-period lag remains positive and statistically significant, with an estimated magnitude of approximately 0.010-0.012. Compared with the contemporaneous effect, the lagged coefficients are substantially larger, indicating that the resilience-enhancing effect of the BRI accumulates over time rather than materializing immediately upon policy participation.

To further examine whether the policy effects differ across countries with different participation trajectories, the sample is divided into early BRI entrants and late BRI entrants based on the timing of accession. The results reveal pronounced heterogeneity in policy impacts. For early entrants, the estimated BRI coefficients are larger in magnitude and statistically significant across both contemporaneous and lagged specifications, indicating a sustained and cumulative improvement in trade resilience following participation. In contrast, for late entrants, the estimated effects are generally smaller and less precisely estimated, with several coefficients failing to reach conventional significance levels. This divergence suggests that earlier integration into the BRI framework allows countries to benefit more fully from cumulative institutional coordination, infrastructure connectivity, and trade network integration, while latecomers face a more gradual and uncertain adjustment process.

The dynamic effects of the BRI are further examined using an event-study specification. The estimated coefficients for the pre-treatment periods (D-5 to D-(2) are small in magnitude and statistically insignificant, and the joint test for parallel trends fails to reject the null hypothesis (*p* = 0.211), supporting the validity of the DID identification assumption. In contrast, the post-treatment coefficients exhibit a clear upward trajectory. While the contemporaneous effect (D0) and the first post-treatment period (D1) are not statistically significant, the coefficients from D2 onward become positive and statistically significant, with magnitudes increasing steadily over time and reaching approximately 0.020 in the later post-treatment periods. This pattern is particularly pronounced among early BRI entrants, further reinforcing the presence of cumulative policy effects.

A series of robustness checks confirms these findings. Placebo tests using fictitious treatment years yield statistically insignificant results, and bootstrap standard error estimates produce coefficient signs and magnitudes consistent with the baseline specifications. Together, these results confirm that the estimated effects are not driven by spurious correlations or model-specific assumptions.

Overall, the DID results provide robust causal evidence that the BRI has enhanced copper resource trade resilience among participating countries. Moreover, the heterogeneity across early and late entrants highlights that the policy effects of the BRI are path-dependent and cumulative, rather than uniform across countries.

#### Linking causal effects to structural and spatial synergy

The empirical evidence suggests that the BRI enhances copper resource trade resilience through selective and path-dependent synergy rather than uniform convergence. Specifically, the causal effects identified in Section causal effects of the BRI on copper resource trade resilience indicate that the BRI strengthens resilience primarily by reinforcing structural coordination and spatial agglomeration among early and institutionally capable participants, while its effects on structurally constrained latecomers remain limited.

First, from a temporal perspective, the dynamic DID results are closely aligned with the phased evolution of trade resilience identified in Section temporal evolution of copper resource trade resilience. The event study estimates show that the positive impact of the BRI emerges only after a lag of approximately two periods and continues to intensify thereafter. This gradual accumulation of policy effects corresponds to the post-2013 acceleration and consolidation phases of copper resource trade resilience, indicating that the BRI operates through sustained institutional and trade-related adjustments rather than short-term shocks. The absence of an immediate effect further suggests that resilience enhancement requires time for coordination mechanisms and trade networks to mature.

Second, from a structural perspective, the heterogeneous effects between early and late BRI entrants provide direct evidence that the BRI’s resilience-enhancing impact is conditional on pre-existing structural capacities. Early entrants exhibit larger and more persistent treatment effects, consistent with the findings in Section who leads, who lags, that adjustment and recovery capacity is the dominant contributor to resilience differences. Countries with stronger governance quality, policy consistency, and international engagement are better able to translate BRI participation into sustained improvements in trade resilience, whereas late entrants experience weaker and less stable gains. This pattern indicates that the BRI amplifies existing structural advantages rather than neutralizing them.

Third, from a spatial perspective, the DID results help explain the evolution of spatial clustering patterns identified in Section who aggregates as core, and who falls to the periphery. The intensification of the BRI’s causal effects coincides with the strengthening of positive spatial autocorrelation in trade resilience after 2014. Moreover, the stronger policy impacts among early entrants are spatially concentrated in regions that form stable HH clusters in the LISA analysis, such as East Asia and Central-Eastern Europe. In contrast, countries located in persistent LL clusters display weaker responsiveness to the BRI, suggesting that spatial proximity alone is insufficient to generate effective synergy without complementary institutional and structural conditions.

## Discussion

Against the backdrop of heightened geopolitical uncertainty, supply chain disruptions, and growing concerns over critical mineral security, enhancing the resilience of resource trade systems has become a central challenge for regional cooperation initiatives such as the BRI. Using copper resource trade as a representative case, this study provides an integrated empirical assessment of how trade resilience evolves over time, differentiates across countries, manifests spatially, and responds causally to large-scale policy intervention. By combining composite resilience measurement, structural decomposition, spatial analysis, and causal identification, the findings offer several insights that extend beyond descriptive patterns and carry direct implications for the optimization of BRI-related cooperation. Overall, the results demonstrate that copper resource trade resilience among Belt and Road countries has improved steadily over the past two decades, but this improvement has been accompanied by persistent and widening cross-country differentiation. High-resilience countries have consolidated their advantages, while low-resilience countries remain structurally constrained. Importantly, the causal evidence indicates that the BRI has played a positive role in enhancing trade resilience, yet its effects are selective and path-dependent, reinforcing structural coordination and spatial synergy primarily among early and institutionally capable participants rather than inducing uniform convergence.

### Rethinking the sources of trade resilience under a core-periphery structure

One key contribution of this study lies in clarifying that sustained leadership in copper resource trade resilience is not solely determined by resource endowment or trade scale, but by a broader configuration of institutional and structural capabilities. Countries such as China, Poland, Russia, and several Central and Eastern European economies have consistently occupied high-resilience positions over the past two decades, exhibiting a pronounced “high-position lock-in” pattern. Structural decomposition results show that this stability is primarily driven by adjustment and recovery capacity, including government efficiency, policy consistency, transparency, and active participation in international agreements, rather than by resource abundance alone. This finding challenges a resource-centric interpretation of resilience and underscores the importance of institutional resilience as a foundational element of trade system stability.^45^ Even resource-rich countries may remain vulnerable to external shocks if institutional coordination and governance capacity are insufficient. Conversely, countries with relatively limited resource endowments, but stronger institutional execution and technological upgrading, can maintain resilient trade positions. From the perspective of the BRI, this implies that enhancing resilience cannot rely on expanding trade volumes or infrastructure connectivity alone; it requires sustained institutional embedding and governance alignment to stabilize cross-border trade relationships.

### Differentiated resilience enhancement paths and the limits of uniform policy design

The results further reveal pronounced heterogeneity in resilience enhancement paths among Belt and Road countries. Medium-resilience countries exhibit divergent trajectories; some have successfully transitioned into stable followers or emerging leaders by upgrading technological complexity and institutional tools, while others have stagnated or declined due to persistent governance and coordination constraints. In contrast, low-resilience countries are often locked into disadvantageous positions, facing a combination of weak institutional capacity, limited market integration, and exposure to political or economic instability. These findings highlight a fundamental limitation of uniform policy design within large-scale cooperation initiatives. While the BRI provides a common platform for trade facilitation and infrastructure investment, countries differ substantially in their capacity to absorb and translate policy incentives into resilience gains.^46^^,^^47^ The DID results reinforce this conclusion by showing that early BRI entrants benefit more strongly and consistently from participation, whereas late entrants experience weaker and more uncertain effects. This divergence reflects path dependence in institutional integration and suggests that resilience enhancement requires long-term capacity accumulation rather than short-term policy exposure.

### Spatial polarization, fragmented diffusion, and the nature of synergy under the BRI

From a spatial perspective, the study shows that structural differentiation in trade resilience is increasingly externalized into a core-periphery pattern. High-resilience cores, particularly in East Asia and Central-Eastern Europe, exhibit persistent HH clustering, while low-resilience troughs remain concentrated in parts of West Asia, the Middle East, and South Asia. At the same time, spatial diffusion of resilience advantages is limited and uneven. High-resilience countries often appear as “spatial islands” surrounded by less resilient neighbors, and low-resilience countries rarely achieve sustained upgrading through geographic proximity alone. Crucially, the causal analysis suggests that the BRI has reinforced these spatial patterns rather than neutralizing them. The strengthening of spatial autocorrelation after 2014 coincides with the period in which BRI-induced resilience effects intensify, particularly among early 7entrants. This indicates that BRI-related synergy operates primarily by consolidating existing structural and spatial advantages, rather than by generating broad-based spillovers. In this sense, the BRI enhances synergy in a selective manner: It deepens coordination where institutional and structural foundations already exist, while leaving deeper asymmetries largely intact.

### Policy implications: Aligning BRI optimization with trade resilience enhancement

The findings of this study carry important policy implications for both the optimization of the BRI and the targeted enhancement of copper resource trade resilience. First, policy efforts should move beyond a narrow emphasis on “hard connectivity” toward strengthening institutional and governance-based resilience. The dominance of adjustment and recovery capacity in explaining resilience differences suggests that improving government efficiency, policy consistency, and transparency is critical for stabilizing trade systems. BRI cooperation frameworks should therefore place greater emphasis on institutional alignment, regulatory coordination, and rule-based cooperation in resource trade.^48^ Second, differentiated policy instruments are essential. Given the heterogeneous effects of BRI participation, a “uniform policy design” approach risks amplifying existing disparities. High and medium-resilience countries can be encouraged to deepen cooperation in areas such as technological upgrading, supply chain diversification, and joint risk management. For low-resilience countries, priority should be placed on capacity-building measures, including technical assistance, institutional reform support, and mechanisms that facilitate gradual integration into regional trade networks.^49^^,^^50^ Such differentiation would allow the BRI to function not only as a connectivity platform, but also as a capacity-equalizing framework. Third, spatial coordination mechanisms should be strengthened to address fragmented diffusion. The persistence of HH and LL clusters indicates that spatial proximity alone does not guarantee resilience spillovers. Policymakers should therefore promote cross-regional cooperation arrangements that link high-resilience cores with structurally constrained neighbors through targeted projects, shared governance mechanisms, and coordinated investment strategies in critical minerals.^51^^,^^52^ This could help transform isolated resilience advantages into broader regional stability. Finally, as the BRI enters a phase of high-quality development, monitoring and evaluation frameworks should be redesigned to focus explicitly on resilience outcomes rather than input-based indicators.^53^ Systematic tracking of trade stability, adjustment capacity, and structural upgrading would improve the precision of policy interventions and enhance the initiative’s long-term effectiveness in managing resource security risks.^54^

### Limitations of the study

This study has several limitations that also point to directions for future research. First, we do not construct an explicit non-BRI comparison group; the policy evaluation relies on staggered participation within BRI countries, which strengthens within-sample identification but cannot directly benchmark resilience trajectories against major non-BRI copper-trading economies. Future work could incorporate a matched non-BRI control set or synthetic control type benchmarks to provide a clearer external comparison. Second, although the resilience index is built from a mechanism-based three-capability framework and an integrated objective-weighting scheme, results may still be sensitive to indicator selection, normalization, and cross-country data quality; future research should test alternative indicator sets and higher-resolution trade data to validate mechanisms and trace transmission channels more precisely.

## Resource availability

### Lead contact

Requests for further information and resources should be directed to and will be fulfilled by the lead contact, Jianping Ge (gejianping@cugb.edu.cn).

### Materials availability

This study did not generate new, unique reagents.

### Data and code availability

The data used in this study were obtained from official government statistics and public databases, all of which are cited in this article. This paper does not report original code. Any additional information required to reanalyze the data reported in this paper is available from the lead contact upon request.

## Acknowledgments

This study was supported by grants from the 10.13039/501100001809National Natural Science Foundation of China [grant nos 72274183, 71774149], the Fundamental Research Funds for the 10.13039/501100001313Coventry University [grant nos 292018006, 292017023], and the Beijing Social Science Foundation [grant no 21DTR059].

## Author contributions

Conceptualization, Y.W. and J.G.; data curation and formal analysis, Y.W. and A.Z.; writing-original draft, Y.W.; writing – review and editing, Y.W. and J.G.; validation: J.G.; funding acquisition: J.G.

## Declaration of interests

The authors declare no conflict of interest.

## STAR★Methods

### Key resources table

**Table:** 

REAGENT or RESOURCE	SOURCE	IDENTIFIER
**Software and algorithms**		

STATA 18.0	This paper	https://www.stata.com/manuals/causal.pdf
ArcMap 10.8	This paper	https://desktop.arcgis.com/zh-cn/arcmap/latest/tools/spatial-statistics-toolbox/an-overview-of-the-mapping-clusters-toolset.htm
Entropy Weighting	Chen et al.^55^	https://doi.org/10.1061/(ASCE)NH.1527-6996.0000280
PCA (Principal Component Analysis)	Guo and Lu.^56^	https://doi.org/10.1108/ECAM-04-2020-0262
CRITIC (Criteria Importance Through Intercriteria Correlation)	Yang et al.^57^	https://doi.org/10.1016/j.evalprogplan.2022.102202
RFE-MF (Recursive Feature Elimination-MissForest) algorithm	Hu et al.^58^	https://doi.org/10.1186/s12874-024-02392-2

### Experimental model and study participant details

Omitted as our study does not involve biological models.

### Method details

To ensure a coherent link between measurement, structural interpretation, spatial organization, and policy evaluation, this study follows a four-step empirical strategy: (1) constructing a multidimensional copper resource trade resilience index using an integrated objective-weighting scheme; (2) decomposing cross-country resilience gaps using Shapley values; (3) identifying spatial clustering and local heterogeneity via Moran’s I and LISA; and (4) estimating the causal impact of the BRI on resilience using a multi-period difference-in-differences (DID) design.

First, the calculation of copper resource trade resilience indicators. The Supply Chain Diversification Index is calculated using the inverse Herfindahl-Hirschman Index (HHI),^59^

(Equation 1) $$\text{SCDI}=1-\sum_{i=1}^{N}S_{i}^{2}$$

where *S* represents the share of a country in the export (or import) market. A higher index indicates a greater diversification and higher capacity to withstand shocks. The Product Technological Complexity Index is based on the economic complexity methodology proposed by Hidalgo and Hausmann (2009),^60^ using the product export structure to estimate the technological complexity of copper resource related products (*PRODY*) and the national copper resource products export complexity (*EXPY*).

(Equation 2) $${\text{PRODY}}_{p}=\sum_{c}\left(\frac{X_{\text{cp}}}{\sum_{c}X_{\text{cp}}}\right)\times {\text{GDP}}_{c}$$

where *x*_*cp*_ represents the export value of country *c* for product *p*; *GDP*_*c*_ is the per capita GDP of country *c*.

(Equation 3) $${EXPY}_{c}=\sum_{p}\left(\frac{X_{cp}}{\sum_{p}X_{cp}}\right)\times {PRODY}_{p\;}$$

where *EXPY*_*c*_ denotes the technological complexity of exports for country *c*; ∑_*p*_*X*_*cp*_ is the total export value of all copper resource products from country *c*.

Second, indicator normalization and direction unification. Let *x*_*ijt*_ denote the raw value of indicator j for country i in year t. To ensure that a higher value consistently represents stronger resilience, reverse indicators are first directionally unified. Then, all indicators are normalized using a Min-Max transformation to eliminate scale differences.

(Equation 4) $$Z_{ijt}=\frac{x_{ijt}-\min\left(x_{j}\right)}{\max\left(x_{j}\right)-\min\left(x_{j}\right)},Z_{ijt}\in \left[0,1\right]$$

where *max*(*x*_*j*_) and *min*(*x*_*j*_) are computed over the full sample period to preserve intertemporal comparability.

Third, objective weighting and index aggregation. The entropy weight method is applied to comprehensively measure trade resilience. This method has the advantages of high objectivity and the ability to reflect indicator variability, making it suitable for multi-indicator comprehensive evaluation scenarios.^55^

Set indicator *x*_*ij*_ represents the value of the *j* for the *i* country. After standardization, the information entropy *e*_*j*_ for the *j* is calculated as follows:

(Equation 5) $$e_{j}=-k\sum_{i=1}^{n}p_{ij}\;\ln\left(p_{ij}\right)$$

(Equation 6) $$p_{ij}=\frac{x_{ij}}{\sum_{i=1}^{n}x_{ij}}$$

(Equation 7) $$k=\frac{1}{\ln\left(n\right)}$$

where *p*_*ij*_ denotes the proportion of the *j* for the *i* country, and *k* is the adjustment coefficient. The redundancy *d*_*ij*_=1-*e*_*j*_ is then calculated, from which the weight $q_{j}=\frac{d_{j}}{\sum_{j=1}^{m}d_{j}}$ of each indicator is obtained. The final composite score is given by:

(Equation 8) $$\omega_{j}^{\left(E\right)}=\sum_{j=1}^{m}q_{j}x_{ij}$$

While the entropy method is widely used for objective weighting, it may assign disproportionately large weights to indicators with high dispersion driven by distributional heterogeneity. To mitigate method-specific sensitivity and improve robustness, this study complements entropy weights with PCA (Principal Component Analysis) based variance structure weights and CRITIC (Criteria Importance Through Intercriteria Correlation) weights, and then integrates them into a composite objective weighting scheme.

PCA is employed as an objective weighting approach by exploiting the common variance structure of the standardized indicator set.^56^ Let *Z*_*ijt*_ denote the normalized value of indicator j for country i in year t. PCA decomposes the indicator matrix into orthogonal principal components *PC*_*k*_ with associated explained variance ratios *r*_*k*_ (where $\sum_{k=1}^{m}r_{k}=1$). Following a variance loading contribution approach, the weight of indicator j is constructed using the absolute component *l*_*jk*_ and the explained variance ratios *r*_*k*_. Specifically, the composite contribution score of indicator j is defined as:

(Equation 9) $$s_{j}=\sum_{k=1}^{m}|l_{jk}|r_{k}$$

and the PCA-based indicator weight is obtained by normalization:

(Equation 10) $$\omega_{j}^{\left(P\right)}=\frac{s_{j}}{\sum_{j=1}^{m}s_{j}}$$

In this study, m=10 indicators are included and all m principal components are retained, while the explained variance ratios *r*_*k*_ are used to differentially weight the contribution of each component.

The CRITIC method incorporates both indicator variability and inter-indicator conflict.^57^ The information content of indicator j is defined as:

(Equation 11) $$c_{j}=\sigma_{j}\sum_{k}\left(1-r_{jk}\right)$$

Where σ_j_ is the standard deviation of indicator j, and *r*_*jk*_ is the correlation coefficient between indicators j and k. The CRITIC weight is given by:

(Equation 12) $$\omega_{j}^{\left(C\right)}=\frac{C_{j}}{\sum_{j}C_{j}}$$

Next, to mitigate the sensitivity of any single objective weighting method and improve robustness, the final indicator weight is constructed as the arithmetic average of the three objective weights:

(Equation 13) $$\omega_{j}=\frac{1}{3}\left(\omega_{j}^{\left(E\right)}+\omega_{j}^{\left(P\right)}+\omega_{j}^{\left(C\right)}\right)$$

This integrated weighting scheme balances information dispersion, variance structure, and indicator conflict, providing a more stable basis for resilience measurement.^61^ Finally, the copper resource trade resilience index for country i in year t is computed as the weighted sum of the normalized indicators:

(Equation 14) $${\text{Resilience}}_{\text{jt}}=\sum_{i=1}^{10}{\omega_{j}Z}_{ijt}\;{\text{Resilience}}_{\text{jt}}\in \left[0,1\right]$$

Fourth, Shapley value decomposition of cross-country resilience gaps. To address the question of which factors drive differences in copper resource trade resilience, this study applies Shapley value decomposition from cooperative game theory.^62^ Let G denote the resilience gap between two groups (e.g., a high-resilience group A and a low-resilience group B) defined in the results section:

(Equation 15) $${G=\text{Resilience}}_{A}\;-{\text{Resilience}}_{B}$$

Let M be the set of contributing components to be attributed (either the three capacity sub-indices {*RA*,*AR*,*TG*} or the ten indicators). Define a value function υ(*S*) as the portion of the gap explained when only the subset *S*⊆*M* is allowed to differ between groups. The Shapley contribution of component j is:

(Equation 16) $$\phi_{j}=\sum_{S\subseteq M\setminus \left\{j\right\}}\frac{\left|S\right|!\left(\left|M\right|-\left|S\right|-1\right)!}{\left|M\right|!}\left[\upsilon \left(S\cup \left\{j\right\}\right)-\upsilon \left(S\right)\right]$$

which satisfies the additivity property $\sum_{j\in M}\phi_{j}=G$. In practice, we implement a two-layer decomposition consistent with the index structure: first attributing the gap to the three capacities, and then further attributing the key capacity gap to its underlying indicators. This ensures interpretability and a direct linkage to the constructed resilience mechanism framework.

Fifth, to identify the spatial distribution characteristics of trade resilience, a spatial weight matrix *W* is constructed. The K-nearest neighbor (KNN) method is used to define adjacency relationships, and the Global Moran’s I index is subsequently calculated.^26^ Spatial autocorrelation analysis was conducted using ArcMap 10.8.

(Equation 17) $$I=\frac{n}{\sum_{i=1}^{n}\sum_{j=1}^{n}w_{\text{ij}}}\times \frac{\sum_{i=1}^{n}\sum_{j=1}^{n}w_{\text{ij}}\left(x_{i}-\bar{x}\right)\left(x_{j}-\bar{x}\right)}{\sum_{i=1}^{n}\left(x_{i}-\bar{x}\right)\left(x_{i}-\bar{x}\right)}$$

where *x*_*i*_ is the trade resilience score of the *i* country, $\bar{x}$ is the mean value, and *w*_*ij*_ denotes the adjacency matrix. The values of Moran’s I range from -1 to 1, with values>0 indicating positive spatial correlation and values<0 indicating negative spatial correlation.

Furthermore, Local Moran's I is employed to identify spatial clustering types, expressed as:

(Equation 18) $$I_{i}=\left(x_{i}-\bar{x}\right)\sum_{j=1}^{n}w_{\text{ij}}\left(x_{j}-\bar{x}\right)$$

Based on significance testing and the value relationships of neighboring units, regions are classified into four spatial association types: (1) HH (high-high clustering), where the country has high resilience and neighboring countries also have high resilience, forming a “high-resilience core”; (2) LL (low-low clustering), where both the country and its neighboring countries have low resilience, forming a “resilience trough”; (3) HL (high-low differentiation), where high-performing countries are “islands”, exhibiting high resilience but surrounded by less resilient neighbors and limited collaboration. (4) LH (low-high differentiation), where the country has low resilience and its neighboring countries have high resilience, benefiting from them. The integrated use of these methods aims to reveal the temporal evolution trends, spatial clustering characteristics, and potential regional synergy mechanisms of copper resource trade resilience.

Sixth, multi-period DID for causal identification of the BRI effect. To identify the causal impact of the BRI on copper resource trade resilience under staggered adoption,^48^ we estimate a two-way fixed-effects DID model:

(Equation 19) $${\text{Resilience}}_{\text{jt}}=\alpha +\beta {\text{BRI}}_{\text{it}}+\gamma X_{\text{it}-1}+\mu_{i}+\lambda_{t}+\varepsilon_{\text{it}}$$

where BRI_it_ is an absorbing treatment indicator that equals 1 for country i in years t ≥*T*_*i*_ (the first participation year) and 0 otherwise; X_it-1_ is a vector of one-period lagged controls; μ_i_ and λ_t_ represent country and year fixed effects. Standard errors are clustered at the country level.

To assess dynamic treatment effects and test the parallel trends assumption, we further estimate an event-study specification based on relative time k=t-*T*_*i*_, omitting one pre-treatment period (k=−1) as the reference:

(Equation 20) $${\text{Resilience}}_{\text{jt}}=\alpha +\sum_{k\neq -1}\delta_{k}D_{\text{it}}^{k}+\gamma {\prime X}_{\text{it}-1}+\mu_{i}+\lambda_{t}+\varepsilon_{\text{it}}$$

where $D_{\text{it}}^{k}$=1(t−*T*_*i*_=k). The parallel trends assumption is supported if the pre-treatment coefficients δ_k_ (for k<0) are jointly insignificant. Robustness is evaluated using placebo timing assignments and resampling-based inference (bootstrap/wild bootstrap) as reported in the results section. The estimation of results was conducted using STATA 18.0.

#### Data sources

This study covers 63 countries along the Belt and Road over the period 2004-2023. Among the BRI economies with available annual data coverage over 2004-2023, we retained 63 countries after excluding Afghanistan and Palestine due to severe and persistent data gaps; the observations for China refer specifically to mainland China and exclude Hong Kong Special Administrative Region (SAR), China; Macao SAR, China; and Taiwan, China. All data sources are listed in Table 1, primarily obtained from authoritative databases such as UN Comtrade, the WDI (World Development Indicators), WGI (Worldwide Governance Indicators), IMF (International Monetary Fund), and the WTO (World Trade Organization). Details are provided in the supplemental information. In this study, “copper resources” refer to major traded commodities associated with the copper industry chain, including raw materials, smelted products, intermediate products, and essential chemical materials required for their production and processing. Specifically, this includes Copper Concentrate, Blister, Refined Copper, and the HS codes are shown in Table 2.^63^ To improve data completeness and accuracy, missing values were imputed using the “Recursive Feature Elimination-MissForest” (RFE-MF) algorithm, ensuring data continuity.^58^ This method combines variable importance ranking with random forest-based nonlinear imputation, enhancing robustness and achieving higher accuracy than simple mean, interpolation, or regression methods, ultimately producing a complete panel dataset. The specific algorithm is provided in the Table S7. The data sources for the control variables used in DID are presented in Table 3.

**Table 1:** Data sources of indicators for copper resource trade resilience

Capacity	Indicator	Attribute	Data Source	Weight (Entropy)	Weight (PCA)	Weight (CRITIC)	Weight (Final)
Resistance and Adaptation	Supply Chain Diversification Index	+	UN Comtrade^64^	0.013	0.146	0.154	0.104
	Resource Production Volume	+	MineralsUK^65^	0.490	0.061	0.102	0.218
	Current Account Balance as % of GDP	+	WDI^66^	0.016	0.116	0.084	0.072
Adjustment and Recovery	Government Efficiency Index	+	WGI^67^	0.020	0.127	0.101	0.083
	Policy Consistency Index	+	WGI^67^	0.034	0.139	0.104	0.093
	Government Transparency Index	+	WDI^66^	0.015	0.013	0.047	0.025
	Degree of Participation in International Agreements	+	WTO^68^	0.130	0.174	0.158	0.154
Transformation and Growth	Technology Patent Investment Intensity	+	IMF^69^	0.123	0.071	0.074	0.089
	Product Technological Complexity Index	+	UN Comtrade^64^; WDI^66^	0.023	0.021	0.045	0.029
	Market Expansion Capability Index	+	UN Comtrade^64^	0.136	0.132	0.133	0.133

**Table 2:** Data sources of HS code for copper resource

No	Category	HS Code
1	Copper Concentrate	260300, 260400, 260500, 260700, 260800, 262020, 262030
2	Blister	282550, 282741, 283325, 310310, 310320, 310390, 310410, 310420, 310430, 310490, 310510, 310520, 310530, 310540, 310551, 310559, 310560, 380810, 380820, 380830, 740110, 740120, 740200
3	Refined Copper	740311, 740312, 740313, 740319

**Table 3:** Data sources of variables for copper resource trade resilience

No	Variable	Data Source
1	BRI (Policy Variable)	Nedopil^44^
2	GDP Per Capita	IMF^69^
3	Mineral Resource Dependence	UN Comtrade^64^
4	Trade Freedom	WGI^67^
5	Exchange Rate Volatility	IMF^69^
6	Government Participation	WDI^66^

### Quantification and statistical analysis

This is an observational social-science study based on country -level panel data, no experimental randomization or prospective sample -size estimation was conducted. No biological subjects, animals, cells, or laboratory experiments were involved.

All quantitative and statistical analyses were conducted using a country-year panel dataset covering 63 Belt and Road countries from 2004 to 2023. The main analytical sample for resilience measurement included 1,260 country-year observations, where n represents country-year observations. Copper resource trade resilience was quantified using a multidimensional indicator system and an integrated objective-weighting scheme. Descriptive temporal analyses report the mean, median, average of the top 25%, and average of the bottom 25% of the resilience index, as shown in Figures 4, 5, and 6. Phased evolution was identified using the three-year moving average growth rate and interquartile range thresholds, with Q1, median, and Q3 used as distributional reference points. Spatial statistical analyses were conducted using ArcMap 10.8. Global Moran’s I was used to evaluate overall spatial autocorrelation, and LISA were used to identify local clustering patterns, including HH, LL, HL, and LH types. Statistical significance for spatial autocorrelation was assessed using reported p values, with p<0.10 and p<0.05 used as significance thresholds, as indicated in the figure legends and results section. DID analyses were conducted using Stata 18.0. The causal effect of BRI participation on copper resource trade resilience was estimated using two-way fixed-effects DID models with country and year fixed effects. Control variables were lagged by one period, and standard errors were clustered at the country level. Dynamic event-study specifications were used to assess pre-treatment trends and post-treatment effects. The parallel-trends assumption was evaluated through joint tests of pre-treatment coefficients. Robustness checks included placebo tests using fictitious treatment years and bootstrap standard errors. Statistical significance was defined as p<0.10, p<0.05, and p<0.01, corresponding to ∗, ∗∗, and ∗∗∗, respectively. Full regression coefficients, standard errors, p-value thresholds, and model specifications are reported in Table 1 and the supplemental information.

## Supplemental information

Table S1. Abbreviations of the 63 Countries Along the Belt and Road

Table S2. Scores of Copper Resource Trade Resilience Indicators for 63 Countries Along the Belt and Road From 2004 to 2023

Table S3. The Result of Shapley’s Two-layer Structural Decomposition

Table S4. The Result of Moran’s I

Table S5. The Result of LISA

Table S6. The Result of BRI Effects on Copper Resource Trade Resilience

Table S7. RFE-MF Algorithm
